# Effectiveness of the Use of Augmented Reality in Teaching the Management of Anaphylactic Shock at the Primary Care Level: Protocol for a Randomized Controlled Trial

**DOI:** 10.2196/22460

**Published:** 2021-01-04

**Authors:** Zalika Klemenc-Ketis, Antonija Poplas Susič, Nina Ružić Gorenjec, Špela Miroševič, Uroš Zafošnik, Polona Selič, Špela Tevžič

**Affiliations:** 1 Department of Family Medicine Faculty of Medicine University of Ljubljana Ljubljana Slovenia; 2 Department of Family Medicine Faculty of Medicine University of Maribor Ljubljana Slovenia; 3 Ljubljana Community Health Centre Ljubljana Slovenia; 4 Institute for Biostatistics and Medical Informatics Faculty of Medicine University of Ljubljana Ljubljana Slovenia

**Keywords:** augmented reality, education, emergency medicine, primary health care

## Abstract

**Background:**

Augmented reality (AR) has benefits and feasibility in emergency medicine, especially in the clinical care of patients, in operating rooms and inpatient facilities, and in the education and training of emergency care providers, but current research on this topic is sparse.

**Objective:**

The primary objective is to evaluate the short-term and long-term effectiveness of the use of AR in the treatment of patients with anaphylactic shock. The secondary objectives are to evaluate the safety in the treatment of patients with anaphylactic shock, evaluate the short-term and long-term effectiveness of stress management in this process, and determine the experiences and attitudes towards the use of AR in education.

**Methods:**

The study will be conducted in 3 phases. In the first phase, we will develop and test the scenario for simulation of anaphylactic shock and the evaluation scale for assessing the effect of the intervention. In the second phase, a single-blinded, randomized controlled trial will be conducted. In the third phase, the use of AR in teaching the management of anaphylactic shock using focus groups will be evaluated qualitatively. All participants will participate in a 1-day training program consisting of a lecture on emergency care and anaphylactic shock as well as exercises in manual dexterity (aspiration, airway management, alternative airway management, artificial respiration, chest compressions, safe defibrillation, oxygen application, use of medication during emergency care). The test group will also focus on education about anaphylactic shock in AR (the intervention). The main outcome will be the evaluation of the participants' performance in coping with a simulated scenario of anaphylactic shock using a high-fidelity simulator (simulator with high levels of realism) and a standardized patient in an educational and clinical environment. The study will be conducted with primary care physicians.

**Results:**

A scenario for the simulation with a high-fidelity simulator and standardized patient has already been developed. For the time being, we are developing an evaluation scale and starting to recruit participants. We plan to complete the recruitment of participants by the end of December 2020, start the randomized controlled trial in January 2021, and finish 1 year later. The first results are expected to be submitted for publication in 2021.

**Conclusions:**

This will be the first study to evaluate the effectiveness of the use of AR in medical teaching. Specifically, it will be based on a clinical case of anaphylactic shock at the primary care level. With our study, we also want to evaluate the translation of these educational results into clinical practice and assess their long-term impact.

**Trial Registration:**

ISRCTN Registry ISRCTN58047410; http://www.isrctn.com/ISRCTN58047410

**International Registered Report Identifier (IRRID):**

PRR1-10.2196/22460

## Introduction

Augmented reality (AR) is a technology that enhances the user's reality with the help of digital information [1]. It maintains the user's connections with the real world and synthesizes the virtual with the real. It typically involves a headset through which one can view a physical reality that has been expanded or supplemented by computer-generated sensory inputs such as sound, video, and graphics [2]. AR differs significantly from virtual reality (VR), as the latter is completely immersive (ie, the real [external] world is completely blocked by the headsets) [2].

In the health care sector, AR is used in medical training [3-5], for surgical interventions [6,7], in nursing [8], in rehabilitation [1], in emergency medicine [2,9], and as a therapeutic aid [10]. In health education, it is used in a wide range of subjects (eg, surgery, forensic medicine, anatomy, clinical life support, cardiology) [4]. Educators use various devices, such as smart glasses, tablets, and smart watches [8]. Systematic reviews from the field of AR in medical education report that the subject is increasingly researched but still in the early stages. Studies have mainly focused on the development, usability, and first implementation of AR for learning. Perhaps the value of this teaching method lies in its motivational effect, the training of psychomotor skills, and the ability to make the invisible visible [5]. However, designed AR applications lack an explicit pedagogical framework [4], and there is no evidence that these applications are able to transmit information to the user [3]. There are also no clinical studies that support the effectiveness of the AR technologies used [1].

In emergency medicine, AR has benefits and feasibility in the clinical care of patients, in operating rooms and inpatient facilities, and in the education and training of emergency care providers, but current research on this topic is scarce [2]. Previous studies have shown that AR can enable reflection through experience [11] and can be useful in procedural learning [2]. It also appears that AR adds an extra level of realism to simulation learning, which improves learner self-confidence and teamwork [2].

Emergency management is an integral part of primary care. As a primary care provider, primary care workers can be confronted with any type of emergency that requires updated knowledge, communication and manual skills, trained personnel, appropriate equipment and practice organization, and necessary medication. The wide range of symptoms and the rarity of situations make it difficult for primary care staff to keep up to date and be competent in life support [12]. The use of new training methods, such as classroom simulations with 3-dimensional (3D), highly realistic simulators [13] or in situ simulations, can provide comprehensive training in handling medical emergencies and identifying potentially dangerous medical situations that are not part of the daily work of primary care physicians and other health care workers [14].

Despite the growing body of evidence that AR is effective in medical education, some studies failed to confirm this, reporting no significant impact of the use of AR on learning and no differences according to device used (mobile or other) [15].

There are also several disadvantages of AR use for medical education that could be important, such as the possibility of deteriorating human connections; technical problems, which could affect the learning process; lack of privacy during learning; and questionable cost benefit.

The primary objective of this study is to evaluate the short-term and long-term effectiveness of the use of AR in the management of patients with anaphylactic shock. The secondary objectives are to assess the safety in the treatment of patients with anaphylactic shock, evaluate the short-term and long-term effectiveness of stress management in this process, and determine the experiences and viewpoints of participants regarding the use of AR in education.

## Methods

### Study Design and Settings

This is a mixed-methods study, incorporating quantitative and qualitative methodology and operating under a pragmatism paradigm.

The study will be conducted in 3 phases. In the first phase, we will develop and test the scenario for simulation of anaphylactic shock and the evaluation scale for assessing the effect of the intervention. In the second phase, a single-blinded, randomized controlled trial (RCT) will be conducted. In the third phase, a qualitative methodology with focus groups will be used to assess the attitudes and experience regarding AR use in participants.

The study will be conducted in a primary health care setting, partly in a classroom and partly in a clinical setting. The Slovenian Ethics Committee (No. 0120-67/2020/6) approved the protocol.

### Participants and Recruitment

#### Phase One

Up to 10 experts from the fields of family and general medicine, emergency medicine***,*** and internal medicine will participate in the development of the scenario for the simulation with a high-fidelity simulator and for AR as well as the evaluation scale for assessing the effect of the intervention.

#### Phase Two

Family medicine physicians will participate in the study. We will send an invitation to all family physicians in Slovenia via the register of family medicine physicians at the Slovenian Medical Chamber. We plan to recruit 150 participants. They will be randomly divided into test and control groups. Inclusion criteria will be a signed informed consent and willingness to participate. Exclusion criteria will be physical inability to participate in activities, previously experienced side effects of using AR, and heart disease.

#### Phase Three

For the focus groups, participants from the test group will participate. According to the guidance in previously reported studies, we expect to include a minimum of 4 participants and a maximum of 12 participants per group [16-19]. Qualitative data collection will be continued until data saturation is achieved. According to Guest et al [20], more than 80% of all themes are discovered by using 2-3 focus groups, and 90% are found by using 3-6 focus groups. For this reason, we anticipate having 3-6 focus groups; however, if the point of saturation is achieved sooner, fewer focus groups will be performed. If possible, focus groups will be stratified by work experiences (<10 years’ experience, 10-20 years’ experience, >20 years’ experience). We feel that family physicians will be more open when in a group with physicians who have similar experience.

### Procedures and Data Collection

#### Phase One

The simulation scenario (the simulation is presented with a high-fidelity simulator in a learning and clinical environment and for AR) is being developed by the researchers on the basis of the 2015 European Resuscitation Council guidelines and 2015 European Academy for Allergology and Clinical Immunology guidelines. The experts will validate the scenario and thus ensure its validity.

An evaluation scale will be developed to assess the impact of the intervention. The evaluation criteria will be developed based on the 2015 European Resuscitation Council guidelines and 2015 European Academy for Allergology and Clinical Immunology guidelines. These criteria are reviewed by the experts using a 2-step Delphi methodology. In the first step, the usefulness of items for assessment are assessed using grades 1 to 7, where 1 means that the criterion is not useful at all and 7 means completely useful. To be included in the evaluation scale, each criterion has to be given an average score of ≥5 points. In the second stage, the rating scale is re-evaluated by experts who express their agreement with the rating scale. We will aim for a consensus rate of 90%. Through this process, we will obtain the final version of the rating scale [21].

#### Phase Two

**Figure 1:**
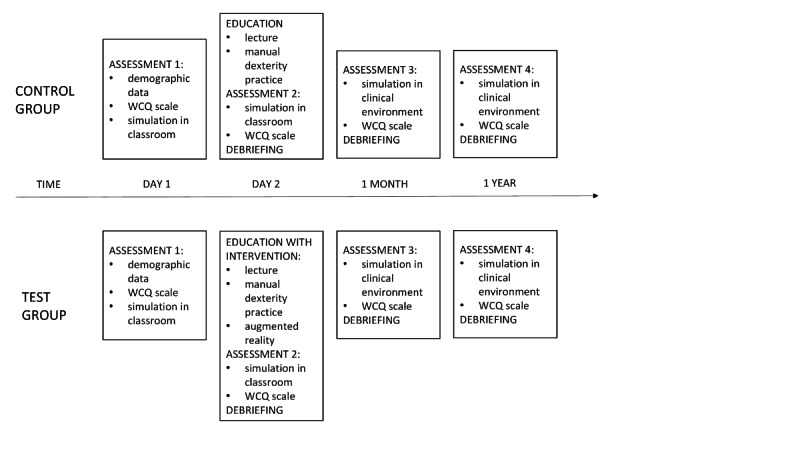
Schematic presentation of the randomized controlled trial in phase two of the study. WCQ: Ways of Coping Questionnaire.

The flow of the study is presented in Figure 1. The study is expected to last for 1 year. Before the intervention, we will assess the baseline characteristics of the participants. All participants will complete the questionnaire on demographic and other data (see Measures). They will also complete the Folkman-Lazarus Ways of Coping Questionnaire (WCQ; please see Measures for more information) [22]. The baseline knowledge, skills, and competencies of the participants regarding the management of a patient with anaphylactic shock will be assessed through a simulation based on the developed scenario using a high-fidelity simulator in a learning environment (a classroom). The participants will perform the simulation in a group of 3; the other 2 members of the group will be educated trainers. They will follow the leadership of the individual participant and not engage in actions until instructed by the participant. The simulation will be video recorded. The recordings will be independently assessed by 3 experts that will not be aware of the participant’s group allocation (blinded), based on the developed evaluation scale. They will harmonize their assessment and produce a single result. This will be in the form of a numeric outcome (primary outcome result) and in a binary form (successful/not successful).

After the intervention (see Intervention), the participants will once again perform the simulation with a high-fidelity simulator and complete the WCQ, which will be followed by a debriefing. We will use a structured tool for debriefing called TALK (Target, Analysis, Learning Points, Key Actions), which is designed to guide structured team self-debriefing after any learning event in clinical environments. It promotes a supportive culture of learning and patient safety [23]. The simulations will be again video recorded, and the recordings will be assessed by experts as already described.

Both groups will be assessed again at 1 month and 1 year after the intervention, using the WCQ scale and simulation. This time, the simulation will be performed in the participant’s workplace and with a standardized patient, followed by a debriefing. The scenario of the simulation will be the same as before. Again, simulations will be video recorded; experts, as already described, will assess the recordings.

The short-term effect will be measured using performance immediately and 1 month after the intervention, while the long-term effect will be measured 1 year after the intervention, which will also be our primary time point.

#### Phase Three

Within 1 month after the intervention, the focus groups consisting of the test group members will be conducted according to qualitative methodology principles. The focus groups will be led by an experienced family physician who has extensive experience in conducting qualitative research and a family physician who will be observing and recording. Participants will be told that the focus groups' primary goal is to explore the experiences, beliefs, and views, providing a comprehensive understanding of the knowledge that participants had with AR in education.

Each focus group will last approximately an hour and will be audio-recorded. Participants will sign an agreement to be recorded. The recordings will be archived for 1 year, then destroyed. Participants will be seated at a round table so they can see each other. Before the group starts, each participant will be provided with a copy of the rules to respect other's opinions, listen to others, and speak in turn. Audio-recordings will be transcribed verbatim.

### Measures

We will record the following demographic and other characteristics of the participants: gender, age, workplace, work period, participation in previous training on anaphylactic shock, being a mentor or tutor, being a teacher, participation in emergency or off-duty care, and previous experience with patients with anaphylactic shock (number of cases).

The WCQ scale provides insight into the processes or strategies of stress management. It contains 66 statements measured on the following scale: 0: none at all; 1: partial; 2: extensive; 3: overwhelming. The statements measure 8 dimensions of stress management: confrontation, distance, self-control, seeking social support, taking responsibility, escape/avoidance, planned problem solving, and positive reassessment. The validity of the construct of the WCQ scale lies in the fact that the research results are consistent with the following theoretical assumptions: (1) Coping involves both problem-oriented and emotionally regulating strategies, and (2) coping is a process. This means that the way stress is handled depends on the demands of the situation and the changes that occur over time [22].

The evaluation scale will consist of several criteria against which we will judge compliance with the guidelines for handling a patient with anaphylactic shock and the safe performance of the procedures. It will also include criteria for assessing the safety of the procedure. Each criterion will be rated as successfully completed or unsuccessfully completed, and the score on the evaluation scale will be the percentage of successfully completed criteria; this will be our primary outcome. Its dichotomous version (which will only be classified as successful if all criteria are successfully completed) will be our secondary outcome. In addition, we will analyze the criteria used to assess the safety of the procedures. Only those participants who meet all safety criteria will be classified as participants who treat the patient in a safe way.

### Intervention

All participants will participate in a 1-day training program consisting of a lecture on emergency care and anaphylactic shock as well as exercises in manual dexterity (aspiration, airway management, alternative airway management, artificial respiration, chest compressions, safe defibrillation, oxygen application, use of medication during emergency care). The test group will receive education on anaphylactic shock in AR. Training with AR is a scenario for the management of a patient with anaphylactic shock. When the head-mounted display is put on, a woman with symptoms of anaphylactic shock that gradually worsen can be seen. Her breathing becomes difficult, an urticarial rash appears, and she says she does not feel well and cannot breathe. When she is asked questions, she answers. The participant must then take the necessary steps to treat this patient correctly; otherwise, the augmented patient will die.

AR intervention represents a combination of standardized and evaluated medical procedures corresponding to a specific medical event (in our case, anaphylactic shock) and digital support id software. Development of an AR intervention consists of (1) defining a medical event, (2) analysis and documentation of crucial parameters (eg, patient type, symptoms, procedures, measurements, medication, equipment involved [eg, monitor, pulse oximeter], decision tree, execution process, and time perspective), (3) defining targeted users or user groups, (4) creation of the use case scenario, (5) defining the expected (un)wanted outcome in the form of standardized results (points achieved, success rate in percentages), (6) software development (environment creation including all 3D assets, animations, sounds, and user interactions), and (7) integration of all elements described into the most believable realistic, holistic experience producing adequate levels of stress in regards to the potential real case scenario. The AR intervention was developed by a group of medical experts (doctor, nurse, and instructor) and information technology experts (user experience engineers, 3D artists, designers, AR developers, and product specialists). The AR intervention will be delivered in the form of software as a service, meaning an application supporting available devices for distributing AR content. Add-ons to the application will be web space for user management and tracking of participant success (pass rate). Trained instructors for emergency medicine education with simulations will deliver the intervention.

### Technical Information

#### Hardware

The primary type of device used for the execution of an AR intervention now is HoloLens, a standalone computer supporting comprehensive 3D rendering, unlimited user movement, voice control, and hand tracking. Any additional controllers will be used. HoloLens supports communication with Internet of Things devices (id sensors or other supportive elements) and the cloud, offering additional computing resources for multiple user experiences (eg, debriefing).

#### Software

The HoloLens uses a Windows Holographic platform. The device’s interface uses gaze input (head tracking), gestures (bloom, air tap, air tap and hold), and voice commands. Three gestures are used to interact with the AR environment: (1) bloom: upward-facing palm, starting with fingertips together, then spreading fingers outward — used for application start-up and closure; (2) air tap (tap and release): with the dorsal aspect of the user’s hand facing them, raising and flexing the index finger (ie, up, down, and up again) in a pinch-like fashion (press and release) — used for selecting an operation; (3) air tap and hold: raising and flexing the index finger to the thumb and motioning the pressed fingers together (press and hold) — used in the user’s 3D space (ie, up, then down) for manipulation of selected objects.

### Ethical Considerations

In this study**,** we will determine the effectiveness of a new teaching method in a controlled environment without health risks. Possible side effects of using AR could be dizziness, headaches, and nausea. Therefore, participants with known similar reactions to similar environments will not be included in the study. Recordings from the study will be used for research purposes only and will be stored on a secure server. The use of a standardized patient in a clinical environment may cause stressful situations. To avoid this, participants are informed immediately before the start of the simulation with the standardized patient that this is a simulation and not a real situation. All participants will be offered a free 1-day training with AR at the end of the study.

### Statistical Analysis

#### Power Calculation

Sufficient sample size to detect a difference between the test and control groups in the score of the evaluation scale (primary outcome) using a 2-tailed independent *t* test was determined using power analysis. For α of .05 and 80% power to reject the null hypothesis of equal group means, when the population mean difference is 10 points (considered clinically relevant) and population SD of both groups is 20 points, a sample size of 128 (64 per group) is needed. The SD of 20 was used based on the results of a study on the use of case-based simulations with high-fidelity mannequins in teaching and retention of emergency management team skills [18]. In that study, the SD of the evaluation scores was <20 for all scenarios. To account for dropouts, we plan to recruit 150 participants.

For the secondary outcome (dichotomous version of the score of the evaluation scale), we performed power analysis using the planned sample size of 128 (64 per group). A chi-square test with α of .05 achieves 80% power to reject the null hypothesis of equal group proportions of successful assessments if the population difference between the group proportions is 21%-25%, where the proportion of successful assessments in the control group is assumed to be between 15% and 40%.

Sample size calculations were conducted using PASS 2019 Power Analysis and Sample Size Software [24].

#### Statistical Methods

We will summarize categorical variables with frequencies and percentages, and we will summarize numerical variables with means and SDs or medians and IQRs in the case of asymmetric distributions. To highlight the differences between the groups at baseline, we will use the chi-squared test or Fisher exact test (if more than 20% of the expected frequencies are below 5) for categorical variables and independent samples *t* test or Mann-Whitney U test (in the case of asymmetric distributions) for numerical variables.

For the comparison of groups after 1 year (primary time point), we plan to use a *t* test for independent samples (or Mann-Whitney U test) for the evaluation scale score (primary outcome) and a chi-squared test (or Fisher exact test) for its dichotomous version (secondary outcome) and for the safety criteria. To compare groups at all time points (right after the intervention, after 1 month, and after 1 year) efficiently in one model, we will use appropriate mixed-effects regression models, which are able to appropriately take into account repeated measurements of the same patient. The power for detecting differences between groups with these models is even higher than with independent *t* tests or chi-square tests that were used in the power analysis.

To evaluate the short-term and long-term effectiveness of coping with stress, only the following dimensions will be included in the analysis: confrontation, distance, self-control, seeking social support, taking responsibility, escape/avoidance, planned problem solving, and positive reassessment. We will sum items to provide each of these scales. For each scale, groups will be compared using a linear mixed-effects regression model.

A *P* value <.05 will be considered as statistically significant.

### Qualitative Analysis

We will perform a thematic analysis following a semantic approach. We intend to get the explicit opinions of the participants on their experiences with education using AR. We do not want to study the underlying assumptions and beliefs that are rooted in the context of the interviews we will perform. The thematic analysis is, according to Guest et al [25], the most useful data analysis technique in capturing the complexity of data within qualitative data and offers a valuable approach for applied research [26]. Thematic analysis is an “organic approach” [27] to coding and generation of the themes that allow for in-depth exploration of the experiences, beliefs, and views, providing a comprehensive understanding of the knowledge that participants had with AR in education. 

The inductive approach in this study will enable researchers to develop a thematic framework emerging from the data (“from the ground up”). A semantic approach will be used since our goal is to explore participants' experiences, beliefs, and views. 

The analysis will be comprised of 6 stages: (1) getting familiar with the data while reading the transcript, (2) generating initial codes, (3) generating themes based on the codes, (4) reviewing initial codes and re(combining) them into previous or new themes, (5) developing and defining names of the themes, and (6) reducing the number of themes into a more manageable set of important themes [27].

We will use NVivo Pro 11 software V.11, 2015 to code the data for the thematic analysis, generate codes and categories, and increase the accuracy of the working methods and result [28]. We will treat data from every stage collaboratively and corroboratively. Multiple researchers from the team will code the data and confirm thematic analysis to ensure that the researcher's perspective does not bias the data's interpretation. This will ensure that the working methods are trustworthy and valid (investigator triangulation). 

## Results

We developed a scenario for simulation with a high-fidelity simulator and standardized patient (Textbox 1). We are currently developing an evaluation scale and starting to recruit participants. We are planning to finish participant recruitment by the end of December 2020, while the main trial will start in January 2021 and finish a year later. The first results are expected to be submitted for publication in 2021.

**Scenario for simulation with a high-fidelity simulator and standardized patient.**

The patient is an otherwise healthy 32-year-old woman with no known history of allergy who received an intramuscular injection of ketoprofen due to back pain. Within minutes, the patient showed signs and symptoms consistent with anaphylaxis, and the participant should quickly take a specific history and physical examination and begin treatment. If the correct diagnosis and treatment are made, the patient will improve. If the participant does not recognize that the patient is in anaphylaxis or is only administering second-line therapy without epinephrine, the patient deteriorates into respiratory arrest with pulseless electrical activity and requires resuscitation according to Advanced Cardiac Life Support (ACLS) guidelines.

## Discussion

### Expected Results

This will be the first study to evaluate the effect of using AR for teaching in an urgent primary health care situation. The main outcome of this study will be the short-term and long-term effectiveness of the use of AR in the training of primary care physicians. We expect that the participants who are trained with AR in addition to the standard training will achieve better results in the treatment of a patient with anaphylactic shock in the simulation compared to other participants.

Several systematic, scoping, and integrative reviews on the use of AR in medical education showed that, while AR technology is growing at a rapid rate, the current quality and breadth of AR research in medical training are insufficient to recommend its adoption into educational curricula [2-4,29]. Existing studies mainly focus on the evaluation of prototypes instead of long-term studies. There is a lack of evidence for the implementation of AR in medical education [8], including training in emergency care [9], even though some studies demonstrated its effectiveness [2,11]. Therefore, we expect that our study will fill the gap on the effectiveness of AR in medical training and provide new insights into its short-term and long-term effects.

Our additional outcome will be the evaluation of safety in the treatment of patients with anaphylactic shock. We expect that participants trained with AR will treat their patients more safely than participants who are not trained with AR. With the use of AR in teaching, we also reach participants who have never seen a patient with anaphylactic shock before. Training with AR enables comprehensive training in dealing with medical emergencies on the one hand and in recognizing potentially dangerous medical situations on the other. This approach also enables us to determine the quality of work and identify potential safety risks in the treatment of patients. We will also evaluate the short-term and long-term effectiveness of stress management in dealing with patients with anaphylactic shock. Stress is widely present in medicine, particularly when dealing with urgent situations [10]. As stress hampers the ability to perform work safely and to achieve a high standard of quality [4], it is important that doctors are educated early on how to manage stress [10]. With AR simulations, we can replicate real patients to reflect real situations in the clinical environment [12] and compare the knowledge of different teams. This provides a safe way to learn how to deal with difficult, unusual, or serious clinical situations. The scenarios are standardized and at the same time flexible, which allows for adaptation to the level of competence of the trainees. The training process is uniform and standardized, which promotes a high quality of learning and does not require years of exposure at accident sites. Realistic, stressful scenarios using highly realistic simulations promote simulated learning, where primary health care teams can interact with patients. Participants learn how to deal with stress during a simulation and manage the patient independently. They can safely explore their feelings and fears and learn how to face and overcome them [13].

With the qualitative part of the study, we want to find out about the experiences with this kind of training, the attitudes toward this teaching method, and suggestions for improvement. We also expect to identify the strengths and limitations of this novel and innovative teaching approach.

### Methodology

The main methodology of this study will be a single-blinded RCT using the AR intervention. We chose AR technology over VR technology because AR allows virtual presence to be blended into the users’ reality with minimal interference. Therefore, we will create a more realistic environment compared to VR, which will allow the training of users in their working environment.

In addition, we will use a qualitative methodology. With such a methodology, we want to prove the validity of our AR application for education or training of medical professionals. According to Barsom et al [3], it is important to focus on 5 levels of validity. Face validity (ie, the degree of similarity between the AR application and the training construct) will be evaluated with focus groups. Content validity (ie, the degree to which the content of the AR application covers the dimensions of the medical content) will also be evaluated with focus groups. The other 3 levels will be assessed through the RCT: (1) construct validity (ie, inherent differences in outcome between experts and novices on outcome parameters relevant to the educational construct), (2) concurrent validity (ie, concordance of the subject's outcome parameters using AR compared to the outcome parameters of an established instrument or method that is assumed to measure the same educational construct), and (3) predictive validity (ie, the degree of agreement between the outcome parameters of AR and the respondent's performance goals, which are supposed to be similar in reality). With the qualitative part of the study, we also want to address the possible downsides of the use of AR in medical education that have already been reported in previous studies [15].

The evaluation of performance will be conducted with a simulation, first in the training environment with a high-fidelity simulator and later in the clinical environment with a standardized patient. With such methodology, we will meet the criteria for translational research [5] using 3 stages: (1) evaluation of performance in the educational environment, (2) evaluation in the clinical environment, and (3) evaluation in health care, community involvement, and prevention services. This study will show the possibility of using AR education for actual clinical practice.

There are some drawbacks of simulation in health care. Simulation relies on space, time, equipment, and skilled human resources. Setting up and running the simulation can be expensive [30]. The simulation center where the study will be conducted is an already established and active center with many simulation trainings conducted over previous years [14]. Therefore, we are certain that these drawbacks will be handled appropriately. Another problematic issue in simulation might be the need for an adaptation period for students to perform the simulation. It is mainly during the second simulation that a student will really start to be able to adapt and treat the patient simulator more realistically [30]. We are aware that this could affect the results of our study, but given the fact that all participants will have the same conditions, we think that this drawback will also be handled appropriately.

In education at all levels and in all fields, studies usually focus on short-term results, assessing the outcome during or immediately after the time when the education took place. Unfortunately, it is true that mastery demonstrated during or immediately after learning can easily be lost in the weeks and months that follow without continued practice [6]. Therefore, in this study, we have chosen to evaluate the short-term and long-term effects by assessing performance immediately after the intervention and 1 month after, given that short-term performance is a good predictor of performance over longer periods of time, and 1 year after the intervention (long-term effect). The long-term preservation of knowledge is the most important, as it is an indicator of permanent memory.

### Limitations

A limitation of this study could be a biased sample, as it is possible that only participants with a high level of interest and motivation will enroll in training courses. There could also be discontinuation of the study, as some participants might demonstrate poor performance results and may not want to participate anymore. To avoid this, the size of our initial sample will account for dropouts during the study. During the study (between the intervention and evaluations after 1 month and 1 year), participants may encounter a patient with anaphylactic shock in their clinical practice or attend training on the subject. This could affect their performance.

### Conclusions

This will be the first study to evaluate the effectiveness of the use of AR in teaching medicine based on a clinical case of anaphylactic shock at the primary care level. With this study, we will evaluate the implementation of AR-related educational results in clinical practice. We will also be able to assess their long-term impact. This study will also serve as a basis for other research in the field of training with AR.

## Abbreviations

3D: 3-dimensional
ACLS: Advanced Cardiac Life Support
AR: augmented reality
RCT: randomized controlled trial
TALK: Target, Analysis, Learning Points, Key Actions
VR: virtual reality
WCQ: Ways of Coping Questionnaire

## References

[1] Eckert M, Volmerg JS, Friedrich CM (2019). Augmented Reality in Medicine: Systematic and Bibliographic Review. JMIR Mhealth Uhealth.

[2] Munzer BW, Khan MM, Shipman B, Mahajan P (2019). Augmented Reality in Emergency Medicine: A Scoping Review. J Med Internet Res.

[3] Barsom EZ, Graafland M, Schijven MP (2016). Systematic review on the effectiveness of augmented reality applications in medical training. Surg Endosc.

[4] Zhu E, Hadadgar A, Masiello I, Zary N (2014). Augmented reality in healthcare education: an integrative review. PeerJ.

[5] Kamphuis C, Barsom E, Schijven M, Christoph N (2014). Augmented reality in medical education?. Perspect Med Educ.

[6] Edström E, Burström G, Nachabe R, Gerdhem P, Elmi Terander A (2020). A Novel Augmented-Reality-Based Surgical Navigation System for Spine Surgery in a Hybrid Operating Room: Design, Workflow, and Clinical Applications. Oper Neurosurg (Hagerstown).

[7] Bertolo R, Hung A, Porpiglia F, Bove P, Schleicher M, Dasgupta P (2020). Systematic review of augmented reality in urological interventions: the evidences of an impact on surgical outcomes are yet to come. World J Urol.

[8] Wüller H, Behrens J, Garthaus M, Marquard S, Remmers H (2019). A scoping review of augmented reality in nursing. BMC Nurs.

[9] Balian S, McGovern SK, Abella BS, Blewer AL, Leary M (2019). Feasibility of an augmented reality cardiopulmonary resuscitation training system for health care providers. Heliyon.

[10] Chicchi Giglioli IA, Pallavicini F, Pedroli E, Serino S, Riva G (2015). Augmented Reality: A Brand New Challenge for the Assessment and Treatment of Psychological Disorders. Comput Math Methods Med.

[11] Zhu E, Lilienthal A, Shluzas LA, Masiello I, Zary N (2015). Design of Mobile Augmented Reality in Health Care Education: A Theory-Driven Framework. JMIR Med Educ.

[12] Ramanayake RPJC, Ranasingha S, Lakmini S (2014). Management of emergencies in general practice: role of general practitioners. J Family Med Prim Care.

[13] Ventola CL (2014). Medical Applications for 3D Printing: Current and Projected Uses. P T.

[14] Klemenc-Ketis Z, Zafošnik U, Poplas Susič A (2020). An innovative approach to educating primary health care teams about medical emergencies. Educ Prim Care.

[15] Gerup J, Soerensen CB, Dieckmann P (2020). Augmented reality and mixed reality for healthcare education beyond surgery: an integrative review. Int J Med Educ.

[16] Krueger RA, Casey MA (2009). Focus groups: a practical guide for applied research.

[17] Kitzinger J (1995). Qualitative research. Introducing focus groups. BMJ.

[18] Kennedy Joshua L, Jones Stacie M, Porter Nicholas, White Marjorie L, Gephardt Grace, Hill Travis, Cantrell Mary, Nick Todd G, Melguizo Maria, Smith Chris, Boateng Beatrice A, Perry Tamara T, Scurlock Amy M, Thompson Tonya M (2013). High-fidelity hybrid simulation of allergic emergencies demonstrates improved preparedness for office emergencies in pediatric allergy clinics. J Allergy Clin Immunol Pract.

[19] Hughes D, DuMont K (1993). Using focus groups to facilitate culturally anchored research. American Journal of Community Psychology.

[20] Guest G, Namey E, McKenna K (2016). How Many Focus Groups Are Enough? Building an Evidence Base for Nonprobability Sample Sizes. Field Methods.

[21] McMillan SS, King M, Tully MP (2016). How to use the nominal group and Delphi techniques. Int J Clin Pharm.

[22] Folkman S, Lazarus RS (1985). If it changes it must be a process: Study of emotion and coping during three stages of a college examination. Journal of Personality and Social Psychology.

[23] (2020). TALK: A tool for structured clinical debriefing.

[24] (2020). PASS 2019 Power Analysis and Sample Size Software.

[25] Guest GS, MacQueen KM, Namey EE (2011). Applied thematic analysis.

[26] Braun V, Clarke V (2014). What can "thematic analysis" offer health and wellbeing researchers?. Int J Qual Stud Health Well-being.

[27] Clarke V, Braun V (2016). Thematic analysis. The Journal of Positive Psychology.

[28] Pope C, Ziebland S, Mays N (2000). Qualitative research in health care. Analysing qualitative data. BMJ.

[29] Tang KS, Cheng DL, Mi E, Greenberg PB (2020). Augmented reality in medical education: a systematic review. Can Med Educ J.

[30] Alinier G (2013). Thesis: Effectiveness of the Use of Simulation Training in Healthcare Education. University of Hertfordshire.

